# Effects of Obesogenic Diet Consumption During Pregnancy and Lactation on the Oxidative Balance and Hepatic Metabolism of Female Rats

**DOI:** 10.3390/nu17132219

**Published:** 2025-07-04

**Authors:** Gizele Santiago de Moura Silva, Deisiane de Araújo Correia, Wellington de Almeida Oliveira, Talitta Arruda Lima, Letícia da Silva Pachêco, Osmar Henrique dos Santos Junior, Reginaldo Correia da Silva Filho, Francisco Carlos Amanajás de Aguiar Júnior, Diogo Antônio Alves de Vasconcelos, Claudia Jacques Lagranha, Sandra Lopes de Souza, Mariana Pinheiro Fernandes

**Affiliations:** 1 Graduate Program of Nutrition, Federal University of Pernambuco, Recife 55670-901, PE, Brazil; gizele.santiago@ufpe.br (G.S.d.M.S.); talitta.ricarlly@ufpe.br (T.A.L.); leticia.pacheco@ufpe.br (L.d.S.P.); diogo.vasconcelos@ufpe.br (D.A.A.d.V.); sandra.lsouza2@ufpe.br (S.L.d.S.); 2 Laboratory of Biochemistry and Exercise Biochemistry, Department of Physical Education and Sports Science, Federal University of Pernambuco, Vitória de Santo Antão 55608-680, PE, Brazil; deisiane.correia@ufpe.br (D.d.A.C.); wellington.almeidaoliveira@ufpe.br (W.d.A.O.); osmar.santosjunior@ufpe.br (O.H.d.S.J.); reginaldo.correia@ufpe.br (R.C.d.S.F.); claudia.lagranha@ufpe.br (C.J.L.); 3 Graduate Program of Nutrition, Physical Activity and Phenotypic Plasticity, Federal University of Pernambuco, Vitória de Santo Antão 55608-680, PE, Brazil; 4 Graduate Program of Biochemistry and Physiology, Federal University of Pernambuco, Recife 55670-901, PE, Brazil; 5 Graduate Program of Neuropsychiatry and Behavioral Sciences Graduate Program, Federal University of Pernambuco, Recife 50670-901, PE, Brazil; 6 Professional Master Program in Biology Teaching, Federal University of Pernambuco, Recife 55670-901, PE, Brazil; francisco.amanajas@ufpe.br

**Keywords:** oxidative stress, nutritional insult, inflammation, lipid metabolism, REDOX state

## Abstract

Background/Objectives: Diets high in simple carbohydrates and saturated fats, commonly consumed in Westernized countries, have been linked to a greater predisposition to metabolic disorders, which are partly attributed to oxidative stress. This study aimed to investigate the impact of an obesogenic diet consumed during the pregnancy and lactation periods on hepatic metabolism and REDOX balance in rats. Methods: Sixteen pregnant Wistar rats were divided into two groups: control (CD), which received a vivarium diet, and obesogenic (OD), which received an obesogenic diet (high-fat diet plus condensed milk), from early pregnancy to late lactation. Thirty-six hours after weaning, the rats were euthanized, and blood, adipose tissue, and liver samples were collected for analysis. Results: These results demonstrate that exposure to an obesogenic diet during pregnancy and lactation in rats leads to adverse changes in hepatic metabolic, inflammatory, and REDOX balance. This experimental animal model serves as a valuable tool for investigating the mechanisms of metabolic dysfunction associated with diets that mimic human eating habits. However, it is essential to note that these findings pertain to an experimental model and therefore require validation in clinical studies to confirm their relevance and applicability in human health. Conclusions: The consumption of an obesogenic diet during pregnancy and lactation in rats induces adverse alterations in hepatic metabolic, inflammatory, and redox homeostasis. This animal model helps investigate the mechanisms of metabolic dysfunctions associated with human dietary habits. However, these findings still need to be confirmed in clinical studies to verify their relevance in humans.

## 1. Introduction

The increase in the prevalence of overweight and obesity has been considered a global pandemic in recent years [1]. The proportion of women of reproductive age who are overweight and obese has increased, as has the incidence of gestational obesity [2,3] and increased risks of obstetric and neonatal complications [4]. Maternal obesity is a significant risk factor for hyperglycemia, gestational diabetes, and metabolic dysfunction-associated steatosis liver disease (MASLD) [5,6,7,8], in addition to affecting breastfeeding [9] and impacting neonatal development and growth [10].

The consumption of an obesogenic diet increased among women during the maternal period [11]. Obesogenic diets are characterized by high saturated fat and sugar content, which induce increased adiposity, hyperlipidemia, and inflammation [12,13]. The combination of a diet high in fat and simple carbohydrates, such as condensed milk, is used in experimental models to simulate Western dietary patterns and induce maternal obesity and metabolic dysfunctions due to energy overload [14,15]. Lean et al. observed that rodents fed an obesogenic diet for three weeks before and during pregnancy exhibited excessive liver fat accumulation and increased adiposity after weaning [16]. The exposure of rats to an obesogenic diet during lactation predisposes their offspring to the development of metabolic syndrome in adulthood [14,17]. The consumption of a high-fat, high-sugar diet during pregnancy and lactation periods in mice also caused moderate/severe hepatic steatosis in adult pups fed with a control diet after weaning [15].

The intake of a high-fat diet increases the uptake of fatty acids and lipogenesis, thereby decreasing lipid oxidation and promoting the accumulation of triglycerides in the liver [18]. Studies have shown that non-pregnant rats fed a high-fat diet developed hepatic steatosis, cellular damage, liver inflammation, and fibrosis [19,20]. Hepatic diseases are also associated with the accumulation of cellular injuries caused by the dysregulation of REDOX homeostasis, which can result from an increased production of reactive oxygen species (ROS) and decreased antioxidant defense enzymes such as superoxide dismutase (SOD), glutathione peroxidase (GPX), and catalase (CAT) enzymes, resulting in cellular oxidative stress [21]. Miranda et al. observed that a maternal high-fat diet introduced 8 weeks before mating and during pregnancy and lactation in rats caused a decrease in the antioxidant enzymes CAT, GPX, and SOD in the livers of male and female offspring in adulthood [22].

Increased ROS generation, produced by oxidative stress, activates hepatic stellate cells, increases the expression of pro-inflammatory cytokines such as tumor necrosis factor-alpha (TNF-α), interleukin-6 (IL-6), and interleukin-1 (IL-1), and triggers apoptosis and liver fibrosis [23]. In mice fed a high-calorie diet during pregnancy and lactation, the development of non-alcoholic steatohepatitis and increased levels of TNF-α were observed, indicating a pro-inflammatory state. Additionally, these mice exhibited increased concentrations of malondialdehyde (MDA) and decreased catalase activity in the liver of their mothers [24].

Most studies using animal models with diets high in fat and sugar have focused on long-term exposure (between 4 and 14 weeks before pregnancy) in established maternal obesity, examining the effects of the maternal diet on offspring [25,26]. However, few studies have demonstrated the impact of an obesogenic diet over a short period, such as during pregnancy and lactation in rats (6 weeks), on maternal health. It is essential to understand the effects of consuming foods with low nutritional quality in mothers who change their eating habits during pregnancy and lactation, as this can have detrimental metabolic effects, even if the mother has been eating a healthy diet before pregnancy. Therefore, we hypothesize that short-term exposure to an obesogenic diet during critical periods of development is sufficient to induce hepatic metabolic dysfunctions characterized by increased lipogenesis and impaired maternal REDOX balance. Thus, this study aimed to investigate the lipogenic effects on hepatic metabolism and REDOX balance in rats that fed an obesogenic diet during pregnancy and lactation.

## 2. Materials and Methods

### 2.1. Animals and Experimental Design

The experiments were performed after approval by the Ethics Committee for Animal Research of the Federal University of Pernambuco-UFPE (approval protocol no. 0061/2019). Sixteen female Wistar rats from the Department of Physiology at UFPE, aged between 80 and 85 days, were used in this study. The animals were housed in an experimental vivarium at a temperature of 22 °C ± 2 °C, with a 12 h light and 12 h dark cycle. The estrous cycle of the rats was monitored, and then they were placed to mate at a ratio of two females to one male. Breeding males were aged between 150 and 200 days. Pregnancy was confirmed using vaginal smear microscopy [27]. After pregnancy detection, the rats were divided into a control diet group (CD) (*n* = 8 animals) that received a Presence^®^ vivarium diet and an obesogenic diet group (OD) (*n* = 8 animals) that received a diet high in lipids and carbohydrates (Table 1). The vivarium diet consists of 10.9% lipids, 28.3% protein, and 60.8% carbohydrates. The obesogenic diet consisted of a high content of saturated fatty acids, adapted from the Westernized diet composition previously described by Ferro Cavalcante et al. [28] and Oliveira et al. [29], with 31.99% of energy coming from fats, 20.18% from proteins, and 47.82% from carbohydrates, prepared in the technical and dietetics laboratory of the Academic Center of Vitória de Santo Antão-UFPE, in addition to supplementation with condensed milk (Italac) (São Paulo, Brazil), with 17.7% from lipids, 9.8% from proteins, and 72.3% from carbohydrates (% values in kcal). Condensed milk was offered separately from the high-fat diet in a porcelain pot installed in the cage. The diet was offered based on pregnancy detection and throughout the pregnancy and lactation period. The pups were weaned at 21 days of age and, after 36 h, the mothers were euthanized by guillotine.

### 2.2. Evaluation of Food Consumption

Food consumption was evaluated daily during the pregnancy and lactation periods. The consumption of feed and condensed milk was measured by the difference between the amount offered and rejected. The porcelain pot containing the condensed milk of each rat was weighed at the beginning of the experiment. The weight of the pot was subtracted during the weighing of consumption, leaving the weight of the condensed milk. A precision digital electronic scale, Vonder, with a maximum capacity of 10 kg, was used to weigh the diet. The bromatological analysis of the diet was performed, and the amount of kcal consumed weekly was determined.

### 2.3. Measurement of Murinometric Parameters, Body Mass, and Hepatic and Adipose Tissue Mass

The waist circumference, naso-anal length, and Lee index were evaluated one day after weaning the pups. Lee’s index was calculated by the cube root of body weight (g) per naso-anal length (cm) and multiplying the result by 1000 [30]. Body mass was assessed every 7 days during the pregnancy and lactation periods. The mass of liver tissue and abdominal and visceral adipose tissue was measured after the euthanasia of the animals. A precision digital scale, Marte (with a capacity of 1010 g and weighing sensitivity of 0.01 g), was used.

### 2.4. Serum Biochemical Profile

Blood samples were collected during euthanasia and conditioned in tubes without anticoagulant, centrifuged at 1400× *g* for 10 min to obtain serum. The supernatant was removed using a pipette and transferred to an Eppendorf tube to perform glucose, total cholesterol, triglycerides, and HDL cholesterol analyses using Labtest^®^ colorimetric kits (Labtest Diagnóstica S.A., Lagoa Santa, Minas Gerais, Brazil).

### 2.5. Triglyceride and Total Cholesterol Levels in the Liver

The liver (100 g) was placed in 1 mL of methanol and homogenized. Then, 2 mL of chloroform was added to each test tube and left overnight in a water bath at 37 °C. Next, 1 mL was added to the organic phase of the samples, which was a solution containing 60% butanol and 40% of a 2:1 mixture of Triton and methanol [31]. After that, triglycerides and total cholesterol levels were measured using Labtest^®^ colorimetric kits.

### 2.6. Oral Glucose Tolerance Test (OGTT)

The OGTT assessment took place on the last day of lactation, following a 12 h fast. Blood samples were collected from cuts on the tip of the animal’s tail; the first blood sample was collected at time zero. Then, 50% glucose solution (Equiplex Pharmaceutical Limited, Aparecida de Goiânia, Brazil) was administered by gavage at a dose of 2 mg/g of body weight. The glycemia of the blood samples was measured using a G-TECH Lite glucometer, at 15, 30, 45, 60, and 120 min after administration of the solution [32].

### 2.7. Preparation of Liver for mRNA Expression

Total RNA was extracted from the livers using the Trizol reagent and the guanidine isothiocyanate method [33] according to the manufacturer’s instructions (Invitrogen, Carlsbad, CA, USA). Then, the RNA pellets were washed in 75% ethanol and centrifuged at 7500× *g* for 5 min at 4 °C, air-dried and dissolved in ultrapure water treated with DEPC. RNA quantification was performed using a NanoDrop 2000 spectrophotometer (Thermo Scientific, Waltham, MA, USA), and purity was assessed by the absorbance ratio at 260/280 nm [34]. Subsequently, real-time polymerase chain reactions (RT-PCR) were performed using β2-microglobulin (β2M) as the normalizing gene: Interleukin 1 beta (IL1β) and tumor necrosis factor-alpha (TNFα) (Table 2) using the SuperScript^®^ III Platinum^®^ SYBR^®^ Green One-Step qRT-PCR kit (Invitrogen, Carlsbad, CA, USA) [35]. Samples (*n* = 6 animals per group) were processed in duplicate, and each target gene’s cycle threshold (Ct) values were normalized to the β2M Ct determined in the identical sample. The relative mRNA expression was determined using the 2-ΔΔCt method [34].

### 2.8. Liver Preparation for Biochemical Analysis

Livers were homogenized in cold buffer (50 mM TRIS, 1 mM EDTA (pH 7.4), 1 mM sodium orthovanadate, and 1.1 mM phenylmethylsulfonyl fluoride-PMSF) with a sampling homogenizer (Tecnal, São Paulo, Brazil). Liver homogenates were centrifuged at 1180× *g* for 10 min at 4 °C, and the supernatants were stored at −80 °C. Protein concentration was determined in the supernatant using Bradford’s method [36] and then utilized for the subsequent biochemical analyses.

### 2.9. Determination of β-Hydroxyacyl-CoA Dehydrogenase (β-HAD) Activity

The (*β-HAD*) activity was determined according to Ito [37]. This enzyme has absolute specificity for the L-isomer of the substrate with hydroxyacyl, converts the hydroxyl at C-3 to a ketone, and generates NADH. Its activity was evaluated in a spectrofluorimeter at a wavelength of 340 nm, a final volume of 0.25 mL. A 50 mM Imidazole solution was prepared, and then a reaction mix (50 mM Imidazole + 12 mM EDTA + 0.18 mM NADH). The reaction mixture with 0.08 mg protein from each sample was incubated for one minute, and 0.1 mM acetoacetyl-CoA was added to initiate the reaction. The reading was performed at an absorbance of 340 nm in a FLUOstar Omega spectrofluorimeter (BMG Labtech, Cary, NC, USA). The results were expressed as nmol/min/mg protein.

### 2.10. Evaluation of Fatty Acid Synthase Activity

Fatty acid synthase (FAS) activity was evaluated following the decrease in absorbance at 340 nm resulting from NADPH oxidation dependent on the addition of malonyl-CoA. Each reaction had 0.10 mg protein, 0.1 M potassium phosphate (pH 7.0), 0.025 mM acetyl-CoA, 0.18 mM NADPH, 3 mM EDTA, 1 mM DTT and 24 mg of albumin, kept at 30 °C. The reaction was initiated by adding the enzyme substrate [38], and the reading was performed in a FLUOstar Omega spectrofluorimeter (BMG Labtech, Cary, NC, USA). The results were expressed as nmol/min/mg protein.

### 2.11. Evaluation of Lipid Peroxidation

Liver protein supernatant (0.3 mg/mL) was used to evaluate the malondialdehyde product (MDA) following the reaction with thiobarbituric acid (TBA). In this method, MDA or MDA-like substances produce a pink pigment with absorption at 535 nm. The reaction was performed by sequentially adding 30% trichloroacetic acid (TCA) and Tris-HCl (3 mM) to the samples, followed by centrifugation at 2500× *g* for 10 min. The supernatant was transferred to a tube, mixed with an equal volume of 0.8% TBA (*v*/*v*), and boiled for 30 min. The absorbance of the organic phase was read at 535 nm in a spectrophotometer, and the results were expressed as mmol per mg of protein [39,40].

### 2.12. Evaluation of Protein Oxidation

The carbonyl content of the protein was evaluated according to Reznick and Packer [41]. A liver protein supernatant (0.3 mg/mL) was used, and 30% TCA was added. The mixture was then centrifuged for 15 min at 1180× *g*. The pellet was suspended in 10 mM 2,4-dinitrophenylhydrazine and immediately incubated in a dark room for 1 h with shaking every 15 min. Afterwards, the samples were centrifuged and washed three times with ethyl/acetate buffer, and the pellet was suspended in guanidine hydrochloride (6 M) and incubated in a water bath at 37 °C for 5 min. The absorbance was read at 370 nm, and the results were expressed as µmol per mg of protein [40,41].

### 2.13. Evaluation of Total Superoxide Dismutase (t-SOD) Activity

The t-SOD activity was evaluated according to the method of Misra and Fridovich [42]. Liver protein supernatant (0.1 mg/mL) was incubated with 880 µL sodium carbonate (0.05%, pH 10.2, 0.1 mM EDTA) at 37 °C, and the reaction was started with 30 mM epinephrine (in 0.05% acetic acid). The kinetics of inhibiting adrenaline auto-oxidation were observed for 90 s at 480 nm. The t-SOD activity was expressed as U/mg protein. One unit of t-SOD corresponds to the amount of protein needed to inhibit the auto-oxidation of 1 μmol of epinephrine per minute [40,42].

### 2.14. Evaluation of Catalase (CAT) Activity

CAT activity was evaluated according to Aebi [43]. The assay consisted of 50 mM phosphate buffers (pH 7.0), 0.300 mM H_2_O_2_, and the liver protein supernatant (0.3 mg/mL). The enzymes kinetics was determined by the absorbance change at 240 nm during 4 min at 30 °C. CAT activity was expressed as U/mg protein. One unit of CAT was defined as the amount of protein required to convert 1 μmol of H_2_O_2_ to H_2_O per minute [40,43].

### 2.15. Evaluation of Glutathione-s-Transferase (GST) Activity

GST activity was evaluated according to Habig [44]. Liver protein supernatant (0.2 mg/mL) was added to 0.1 M phosphate buffer (pH 6.5) containing 1mM-EDTA at 30 °C. The assay was initiated with 1 mM 1-chloro-2.4-dinitrobenzene plus 1 mM reduced glutathione (GSH) and the formation of 2.4-dinitrophenyl-s-glutathione was monitored at 340 nm of absorbance. GST activity was defined as the amount of protein required to catalyze the formation of 1 μmol of 2.4-dinitrophenyl-S-glutathione. The results were expressed as U/mg protein. One unit of GST was defined as the amount of protein required to catalyze the formation of 1 mmol of 2.4-dinitrophenyl-S-glutathione per minute [40,44].

### 2.16. Evaluation of REDOX State (GSH/GSSG Ratio)

GSH levels were measured in a milieu containing 0.1 M phosphate buffer with 5 mM-EDTA (pH 8.0) and liver protein supernatant (0.2 mg/mL). The samples were incubated with 1 mg/mL of o-phthaldialdehyde (OPT) at room temperature (RT) for 15 min, and the fluorescence intensity was assessed at an excitation wavelength of 350 nm and an emission wavelength of 420 nm. To determine the oxidized glutathione (GSSG) levels, liver protein supernatant (0.2 mg/mL) was incubated with 0.04 M N-ethylmaleimide for 30 min at RT, followed by the addition of 0.1 M NaOH buffer up to 0.2 mL. In sequence, similarly to GSH, aliquots were incubated with OPT, and the fluorescence intensity was assessed in the same settings as the GSH assay. The GSH and GSSG values were compared to standard curves, and the REDOX state was calculated as the ratio of GSH/GSSG [45].

### 2.17. Evaluation of Total Thiol (SH) Groups

The measurement of total thiols was based on the reduction of 5,5′-dithio-bis (2-nitrobenzoic acid) (DTNB) by thiols. Liver protein supernatant (0.3 mg/mL) was incubated in the dark with 30 μL of 10 mM DTNB, and a final volume of 1 mL was obtained by the addition of extraction buffer (pH 7.4). The absorbance was read at 412 nm, and the results expressed as M/mg of protein [40,46].

### 2.18. Histology of Hepatocytes

The right lobe of the liver from each animal was collected and fixed in a 10% formalin solution for 24 h. Sections of 4 microns in thickness were cut in the sagittal plane and fixed on microscope slides, followed by staining with hematoxylin and eosin (H&E) to evaluate the morphology and size of hepatocytes. The samples were examined using conventional optical microscopy (Nikon Eclipse E200, São Paulo, Brazil). Images were captured by a TUCSEN USB 2.0 H Series camera and analyzed using Image J Pro v.2.0. and computer software 1.44 (Research Services Branch, US National Institutes of Health, Bethesda, MD, USA). The central region of the right lobe of the liver was analyzed on slides by evaluators blinded to sample identification. Hepatocytes were evaluated for their number, cell length, diameter, and nuclei. For an analysis of the area, perimeter, and diameter of the nucleus and hepatocyte, 1000 cells were selected per experimental group. The number of cells per animal was determined according to the relationship between the number of animals and the minimum number of cells that should be subjected to morphometry. To analyze the number of hepatocytes, two quadrants were selected in each image. The quadrants were added by the software with an area of 1500 µm [40,47].

### 2.19. Statistical Analysis

The analyses were performed using GraphPad Prism software, version 9.0 for Mac (GraphPad, La Jolla, CA, USA). The data passed the normality test (Kolmogorov–Smirnov), confirming their normal distribution and were symmetrical within the Gaussian curve. The data were then subjected to statistical analysis (unpaired Student’s *t*-test). The values were expressed as mean ± standard error of the mean (SEM), and comparisons were considered statistically significant when *p* ≤ 0.05.

## 3. Results

### 3.1. Food Consumption

**Figure 1:**
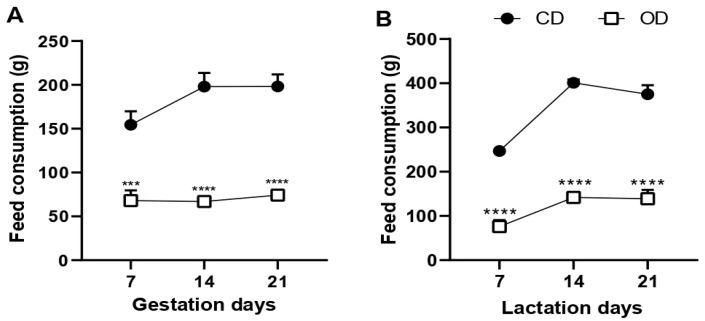
Food consumption in grams (g) of rats fed with an obesogenic diet during pregnancy and lactation. (**A**) Food consumption during pregnancy; (**B**) food consumption during lactation. *** *p* < 0.001; **** *p* < 0.0001, *n* = 5 animals per group. Groups: control diet group (CD); obesogenic diet group (OD).

Our study provided Wistar rats with an obesogenic diet, consisting of a hyperlipidic diet supplemented with condensed milk, during pregnancy and lactation. When evaluating the food consumption of these rats, we observed that the intake of a hyperlipidic diet was lower in the OD group at each week of pregnancy when compared to the ingestion of a vivarium diet in the CD group (7 days: CD = 171.0 ± 10.23, OD = 68.0 ± 11.63 g, 60.25%, *p* = 0.0002; 14 days: CD = 198.0 ± 15.65, OD = 67.0 ± 5.357 g, 66.16%, *p* < 0.0001; 21 days: CD = 198.2 ± 13.72, OD = 74.20 ± 4.306 g, 62.56%, *p* < 0.0001) (Figure 1A). The same result was observed during the lactation period (7 days: CD = 247.6 ± 5.732, OD = 76.40 ± 14.81 g, 69.14%, *p* < 0.0001; 14 days: CD = 401.8 ± 7.719, OD = 142.2 ± 11.55 g, 64.60%, *p* < 0.0001; 21 days: CD = 374.4 ± 20.51, OD = 139.2 ± 19.77 g, 62.91%, *p* < 0.0001) (Figure 1B).

**Figure 2:**
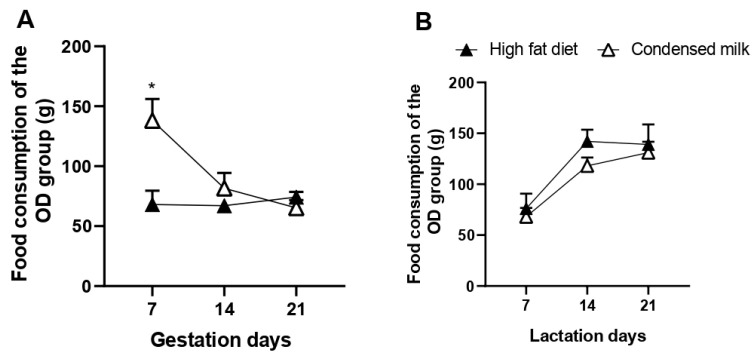
Food consumption in grams (g) of rats from the OD group during pregnancy and lactation. (**A**) Food consumption of the hyperlipidic diet versus condensed milk during pregnancy; (**B**) food consumption of the hyperlipidic diet versus condensed milk during lactation. * *p* < 0.05, *n* = 5 animals per group. Groups: control diet group (CD); obesogenic diet group (OD).

Observing the OD group separately, we noticed that in the first week of pregnancy, there was a higher intake of condensed milk when compared to the intake of a high-fat diet (7 days: high-fat diet = 68.0 ± 11.63 g, condensed milk = 127.0 ± 19.28 g, 86.76%, *p* = 0.0306), but there was no difference in the following weeks of pregnancy (Figure 2A), nor during lactation (Figure 2B).

When evaluating the consumption of macronutrients in the diet, we observed that the rats in the OD group consumed less protein in kcal (CD = 550.6 ± 43.39, OD = 268.0 ± 11.71 kcal, 51.32%, *p* = 0.0002) and more lipids in kcal (CD = 213.1 ± 16.79, OD = 434.9 ± 18.36 kcal, 104.08%, *p* < 0.0001) during the total pregnancy period when compared to the CD group. Carbohydrate consumption and total macronutrient consumption showed no statistical difference between the groups during the entire pregnancy period (Table 3).

During the entire lactation period, the results also showed a decrease in protein intake in kcal (CD = 1025 ± 19.53, OD = 392.0 ± 42.71 kcal, 61.75%, *p* < 0.0001) and an increase in lipid intake in kcal (CD = 396.6 ± 7.55, OD = 674.7 ± 50.52 kcal, 70.12%, *p* = 0.0006) in the OD group compared to the CD group. In addition, the OD group reduced carbohydrate consumption in kcal (CD = 2201 ± 41.95, OD = 1410 ± 108.4 kcal, 35.93%, *p* = 0.0001) during the entire lactation period compared to the CD group. The total intake of macronutrients in kcal was also reduced by (CD = 3623 ± 69.03, OD = 2477 ± 194.8 kcal, 31.63%, *p* = 0.0005) compared to the CD group during the entire lactation period (Table 4).

### 3.2. Body Mass Curve, Adipose and Hepatic Tissue Mass, and Murinometric Parameters

**Figure 3:**
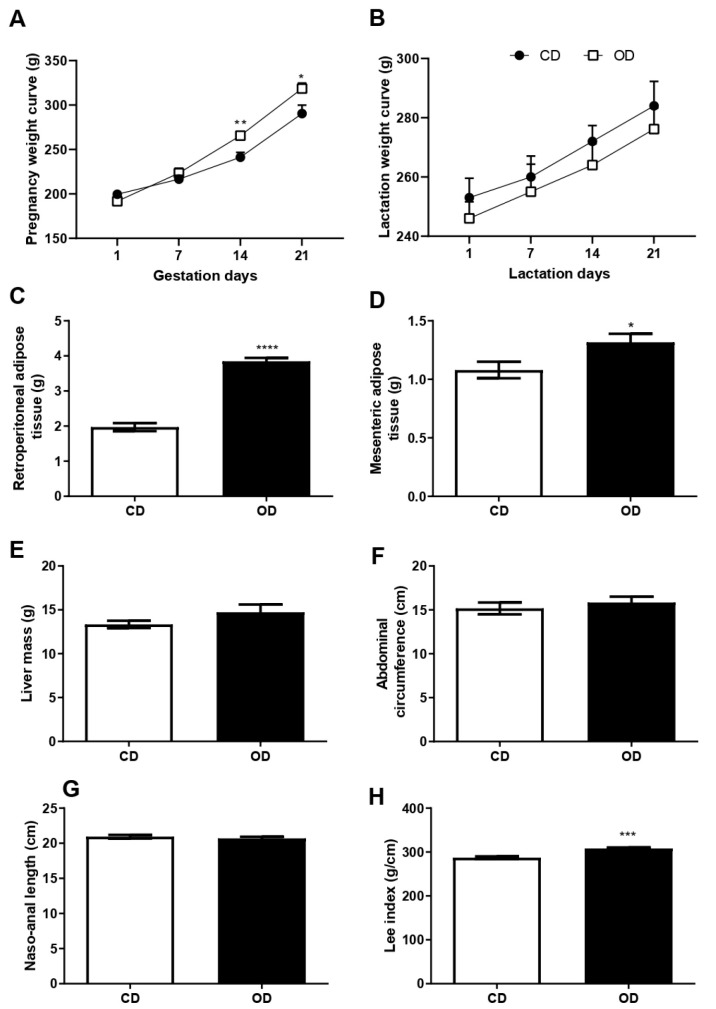
Effects of the obesogenic diet offered to rats during pregnancy and lactation. (**A**) Body mass curve during the pregnancy; (**B**) body mass curve during the lactation; (**C**) mass of retroperitoneal adipose tissue after euthanasia; (**D**) mass of mesenteric adipose tissue after euthanasia; (**E**) liver mass after euthanasia; (**F**) abdominal circumference after lactation; (**G**) nasal length after lactation; (**H**) lee index after lactation. *n*: 5–8 animals per group. Groups: control diet group (CD); obesogenic diet group (OD).

The rats in the OD group showed an increase in body mass in the last two weeks of pregnancy when compared to the CD group, at 14 days (CD = 241.2 ± 5.562; OD = 265.6 ± 2.015 g;10.11%, *p* = 0.0033) and 21 days (CD = 290.4 ± 9.485; OD = 318.6 ± 6.177 g; 9.63%; *p* = 0.0377) (Figure 3A), but there was no statistical difference during lactation (Figure 3B). Retroperitoneal and mesenteric adipose tissue mass increased in the OD group when compared to the CD group, respectively (CD = 1.972 ± 0.1154, OD = 3.847 ± 0.0952 g, 95.08%, *p* < 0.0001) (Figure 3C) and (CD = 1.080 ± 0.0704, OD = 1.317 ± 0.0729 g, 21.94%, *p* = 0.0374) (Figure 3D). Liver issue mass showed no statistical difference between groups (Figure 3E). The murinometric parameters evaluated after lactation showed an increase in the Lee index (CD = 287.0 ± 3.286, OD = 308.2 ± 2.464 g/cm, 7.38%, *p* = 0.0004) (Figure 3H) in the OD group compared to the CD group, however the abdominal circumference and the naso-anal length did not present a significant difference (Figure 3F,G).

### 3.3. Serum and Liver Biochemical Profile

The serum levels of glucose, triglycerides, and total cholesterol were higher in the OD group when compared to the CD group, glucose (CD = 106.3 ± 7.459, OD = 140.2 ± 10.25 mg/dL, 31,89%, *p* = 0.0200); triglycerides (CD = 157.3 ± 19.67, OD = 283.3 ± 24.06 mg/dL, 80.10%, *p* = 0.0037); total cholesterol (CD = 71.41 ± 7.266, OD = 92.40 ± 5.508 mg/dL, 29.39%, *p* = 0.0469). HDL serum levels were lower in the OD group compared to the CD group (CD = 0.03677 ± 0.0004, OD = 0.03422 ± 0.0006 mg/dL, 7.35%, *p* = 0.0080). The levels of triglyceride and total cholesterol in the hepatic tissue also increased in the OD group, respectively (CD = 179.7 ± 8.961, DO = 237.6 ± 18.53 mg/dL, 32.22%, *p* = 0.0226) and (CD = 45.63 ± 3.578, DO = 61.41 ± 1.745 mg/dL, 34.58%, *p* = 0.0042) (Table 5).

### 3.4. Oral Glucose Tolerance Test

**Figure 4:**
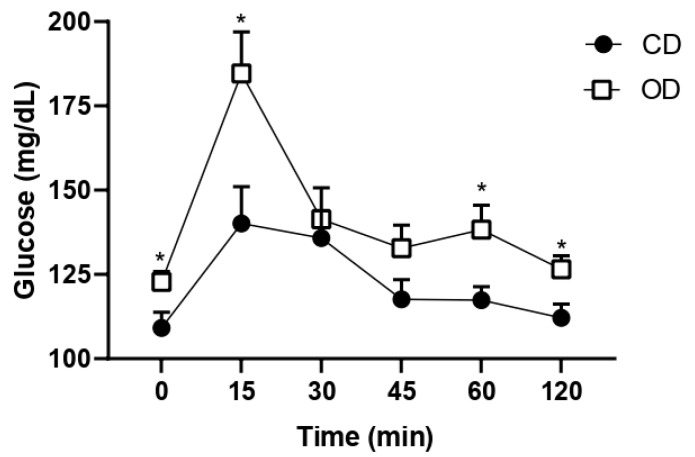
Evaluation of the oral glucose tolerance test in rats fed an obesogenic diet during pregnancy and lactation. The test was performed on the last day of lactation. *n*= 8 animals per group. * *p* < 0.05. Groups: control diet group (CD); obesogenic diet group (OD).

In the OGTT, a significant increase in glucose levels was observed in the OD group at times 0 (pre-glucose administration), 15, 60, and 120 min after the administration of 50% glucose when compared to the CD group, 0 min (CD = 109.1 ± 4.707, OD = 122.8 ± 3.121 mg/dL, 12.55%, *p* = 0.0301), 15 min (CD = 140.1 ± 10.91, OD = 184.6 ± 12.34 mg/dL, 31.76%, *p* = 0.0172), 60 min (CD = 117.4 ± 4.018, OD = 138.3 ± 7.262 mg/dL, 17.80%, *p* = 0.0491), and 120 min (CD = 112.1 ± 4.046, OD = 126.5 ± 4.040 mg/dL, 12.84%, *p* = 0.0248) (Figure 4).

### 3.5. Metabolic Enzymes

**Figure 5:**
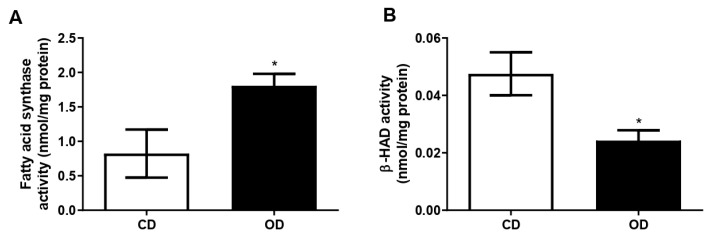
Effect of the obesogenic diet on the activity of metabolic enzymes in the liver of rats. (**A**) Activity of the enzyme fatty acid synthase (FAS) and (**B**) activity of the enzyme β-hydroxyacyl-CoA dehydrogenase (β-HAD)*. n*= 4–5 animals per group. * *p* < 0.05. Groups: control diet group (CD); obesogenic diet group (OD).

The activity of the β-HAD enzyme, the main enzyme responsible for fat oxidation, was evaluated in the liver homogenate. We observed a significant reduction in the activity of this enzyme in the OD group (CD = 0.0475 ± 0.0074, OD = 0.0242 ± 0.0036 nmol/mg of protein, 48.92%, *p* = 0.0230) (Figure 5A). On the other hand, we observed that the FAS enzyme, the enzyme responsible for the synthesis of fatty acids, showed increased activity in the OD group compared to the CD group (CD = 0.8229 ± 0.3489, OD = 1.806 ± 0.1742 nmol/mg of protein, 119.46%, *p* = 0.0307) (Figure 5B).

### 3.6. Histological and Morphometric Analysis of the Liver

**Figure 6:**
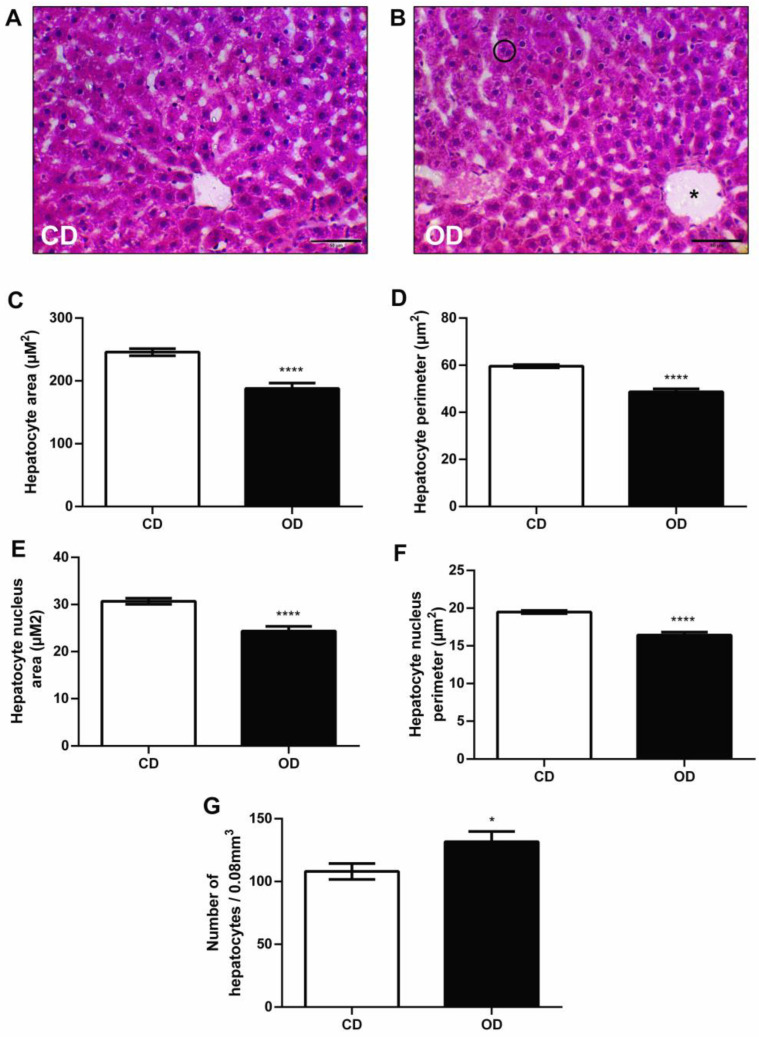
Histological and morphometric analysis of the right lobe of the liver of rats fed an obesogenic diet during pregnancy and lactation. (**A**) Image of hepatocytes at 400× magnification of the CD group; (**B**) image of hepatocytes at 400× magnification of the OD group (circle represents binucleated hepatocytes and the asterisk represents the central vein of the lobule); (**C**) hepatocyte area; (**D**) perimeter of the hepatocyte; (**E**) hepatocyte nucleus area; (**F**) hepatocyte nucleus perimeter; (**G**) number of hepatocytes. *n*= 5 animals per group. * *p* < 0.05; **** *p* < 0.0001. Groups: control diet group (CD); obesogenic diet group (OD).

In a morphometric analysis, we found that the animals in the OD group had a smaller hepatocyte area and perimeter, respectively (CD = 2.458 ± 5.603, OD = 187.9 ± 8.719 µm^2^, 23.55%, *p* < 0.0001; (CD = 59.55 ± 0.6728, OD = 48.70 ± 1.284 µm^2^, 18.21%, *p* < 0.0001) (Figure 6C,D) compared to group CD. The results also showed that there was a smaller nuclear area (CD = 30.68 ± 0.6414, OD = 24.35 ± 0.9966 µm^2^, 20.63%, *p* < 0.0001) and a smaller nuclear perimeter (CD = 19.49 ± 0.2067, OD = 16.43 ± 0.3934 µm^2^, 15.70%, *p* < 0.0001) of hepatocytes in the OD group (Figure 6E,F). Furthermore, we observed a greater number of hepatocytes in group OD (CD = 108.0 ± 6.257, OD = 131.7 ± 8.148 mm^3^, 21.94%, *p* = 0.0241) (Figure 6G). In Figure 6B it is possible to identify the largest number of cells.

### 3.7. mRNA Expression of Inflammatory Markers

**Figure 7:**
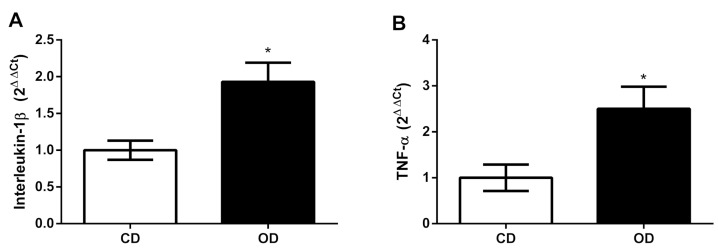
Effect of the obesogenic diet on pro-inflammatory markers in the liver rats. (**A**) Interleukin-1β (IL-1β), and (**B**) Tumor Necrosis Factor-alpha (TNFα). *n* = 6 animals per group, * *p* < 0.05. Groups: control diet group (CD); obesogenic diet group (OD).

An obesogenic diet may exacerbate liver damage by inducing pro-inflammatory cytokines. Thus, we evaluated the gene expression of interleukin-1β (IL-1β) and tumor necrosis factor-alpha (TNF-α) in our liver samples and observed an increase in these cytokines in the OD group, IL-1β (CD = 1.000 ± 0.1300, OD = 1.930 ± 0.2600 2^ΔΔCt^, 93%, *p* = 0.010) and TNF-α (CD = 1.000 ± 0.2900, OD = 2.500 ± 0.4800 2^ΔΔCt^, 150%, *p* = 0.029) (Figure 7A,B).

### 3.8. Pro-Oxidant Markers and Enzymatic and Non-Enzymatic Antioxidant Defenses

**Figure 8:**
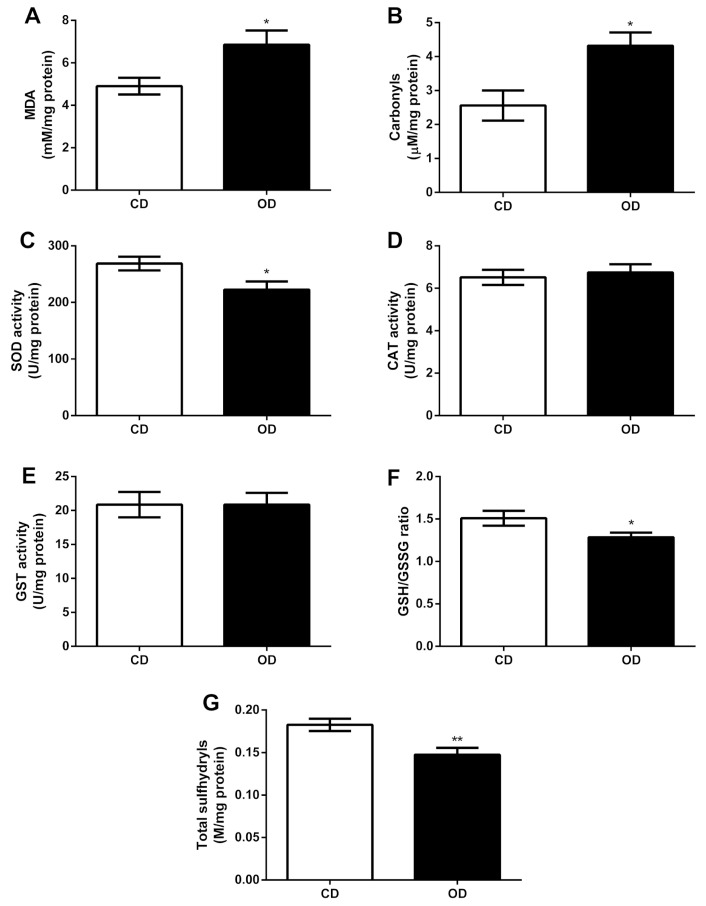
Effect of obesogenic diet on oxidative stress biomarkers and enzymatic and non-enzymatic antioxidant system in rat liver. (**A**) Lipid peroxidation evaluated by the levels of Malondialdehyde (MDA); (**B**) protein oxidation evaluated by carbonyl levels; (**C**) superoxide dismutase (SOD) activity; (**D**) catalase (CAT) activity; (**E**) Glutathione S-transferase (GST) activity; (**F**) REDOX levels measured by the ratio between reduced glutathione (GSH) and oxidized glutathione (GSSG); (**G**) sulfhydryl’s content. *n* = 6–8 animals per group; * *p* < 0.05; ** *p* < 0.01. Groups: control diet group (CD); obesogenic diet group (OD).

We evaluated oxidative stress biomarkers in liver homogenates and observed an increase in lipid (CD = 4.904 ± 0.3959, OD = 6.859 ± 0.6631 mM/mg of protein, 39.86%, *p* = 0.0238) and protein (CD = 2.560 ± 0.4434, OD = 4.320 ± 0.3917 µM/mg of protein, 68.75%, *p* = 0.0117) oxidation induced by the obesogenic diet in the OD group (Figure 8A,B). The antioxidant enzymes SOD, CAT, and GST are responsible for protecting cells against oxidative damage caused by ROS formation. Here, the decrease in SOD activity (CD = 268.7 ± 12.05, OD = 222.6 ± 14.51 U/mg of protein, 17.15%, *p* = 0.0308) is notable (Figure 8C) in the OD group, without differences in CAT and GST activities (Figure 8D,E).

In addition to the enzymatic antioxidant system, ROS-induced damage is also managed by non-enzymatic systems, in which the REDOX state (GSH/GSSG ratio) and total thiol groups represent the main indicators of non-enzymatic capacity. The measurement of these compounds shows that the obesogenic diet decreases the REDOX status (CD = 1.509 ± 0.0876, OD = 1.285 ± 0.0538 ratio, 14.84%, *p* = 0.0499) and total thiol groups (CD = 0.1825 ± 0.0072, OD = 0.1476 ± 0.0082 M/mg of protein, 19.12%, *p* = 0.0098) (Figure 8F,G).

## 4. Discussion

Our study revealed that the maternal consumption of an obesogenic diet, introduced in early pregnancy, resulted in increased body weight during pregnancy, increased adipose tissue, elevated levels of triglycerides and total cholesterol in the liver and serum, and decreased glucose tolerance. We relate the increase in adiposity to the greater consumption of dietary lipids ingested during pregnancy and lactation, in addition to the increased activity of the FAS enzyme and the decrease in lipid oxidation observed due to the lower activity of the β-had dehydrogenase enzyme. The consumption of a diet high in fat and sugar, initiated during pregnancy and continued during lactation, also causes adverse effects on the liver of rats, leading to increased pro-inflammatory cytokines and oxidative stress. Our findings demonstrate the importance of studying the impacts of consuming foods with low nutritional quality during pregnancy and lactation and observing possible adverse effects on maternal lipid metabolism and hepatic REDOX status. Westernized diets, characterized by high levels of saturated fats and sugars, have been identified as two key factors contributing to excess weight during pregnancy [48]. In our study, we found that rats in the OD group consumed a lower amount of the obesogenic diet but had a higher caloric intake from fat in the diet during pregnancy and lactation, as well as a lower caloric intake from protein in both periods. The consumption of calories derived from carbohydrates was only reduced during lactation. These results are similar to those of studies that used obesogenic diets before mating, during pregnancy, and lactation, and observed an increase in fat and sugar consumption, accompanied by a reduction in protein intake, compared to controls [26,49,50].

The obesogenic diet provided to the animals in this study presents a hyperlipidic and hyperglycemic profile, characterized by high levels of saturated fat, primarily from lard, and sucrose, with condensed milk as the predominant source. Although the OD group consumed a smaller amount of dietary grams, the energy values of the obesogenic diet’s components were higher than those of the control diet. Thus, the data from our study show that an obesogenic diet, even in a short period such as pregnancy and lactation, has repercussions on metabolic prejudices for the mother in the OD group, due to the characteristics of the diet and not just the amount ingested. The diet’s composition of macronutrients can influence body fat storage [51]. Due to its caloric density, excess fat in the diet increases the adiposity index and visceral and body fat compared to diets with sugar or controls [52]. Rats in the OD group demonstrated excess weight in the last two weeks of pregnancy compared to the CD group. There was no significant difference in body weight during lactation. However, after lactation, the OD group obtained a greater accumulation of mesenteric and retroperitoneal adipose tissue and a higher Lee index.

These results show that the obesogenic diet predisposes to increased body adiposity and consequently to the development of maternal obesity. During pregnancy, it is normal to consume more food to increase fat mass as a potential energy reserve for the extreme metabolic demands of lactation [53]. However, the higher caloric intake coming mainly from dietary lipids during pregnancy and lactation in this study had a negative impact, inducing an excessive accumulation of body fat in rats, corroborating the study by Qiao et al. (2019), which found that consuming a hyperlipidic diet during pregnancy in rodents increased visceral and subcutaneous fat [54].

The rats in the OD group exhibited increased serum levels of glucose, triglycerides, and total cholesterol, as well as reduced HDL levels, indicating dyslipidemia. These data are like those reported by Li et al. [55], who also observed an increase in glucose, triglycerides, and total cholesterol, along with a decrease in HDL, in rats with maternal obesity induced by a high-fat diet compared to control groups. However, in the study performed by Li et al. [55], the diet was offered before mating, during pregnancy, and during lactation. Dyslipidemia, in conjunction with the findings above, including increased body weight, increased body adiposity, and an elevated Lee index, is associated with the development and progression of metabolic-related fatty liver disease, also known as MASLD. This evidence has been demonstrated in several epidemiological studies [56,57,58]. Furthermore, our results also showed an increase in triglycerides and total cholesterol in the liver of rats that consumed a maternal obesogenic diet, confirming the predisposition to the development of MASLD.

The liver internalizes lipids from the bloodstream, including fatty acids released from white adipose tissue and fatty acids derived from de novo lipogenesis that are used for triglyceride synthesis [59]. Within the liver, insulin action shifts cellular metabolism from energy release and secretion to energy storage [60,61]. Thus, intrahepatocellular triglyceride accumulation can lead to dysregulated insulin signaling, resulting in elevated plasma lipid and glucose concentrations [62]. The homeostatic glucose imbalance results in hyperglycemia and glucose intolerance, which are characteristics of gestational diabetes [63]. In our study, we observed that the OD group remained with elevated glucose levels for longer than the CD group during the OGTT, indicating lower glucose tolerance induced by the maternal obesogenic diet. This result corroborates other studies that have also observed a decrease in glucose tolerance in rats fed an obesogenic diet, specifically during pregnancy [11] or before mating, during pregnancy, and during lactation [64].

The imbalance in hepatic lipid homeostasis between the acquisition and removal of fatty acids results in the abnormal accumulation of lipids, as described in [65,66]. Lipogenesis and fatty acid β-oxidation are the two main processes responsible for lipid balance during liver fat development [66]. In the present study, we observed an increase in FAS activity in the liver of rats in the OD group. This is one of the key enzymes for lipid synthesis, which acts on de novo lipogenesis when there is an excess of carbohydrates in the diet. In contrast, the activity of the β-HAD enzyme was reduced in the livers of rats fed an obesogenic diet, resulting in decreased hepatic fatty acid oxidation. These results justify the increased deposition of triglycerides and cholesterol in the liver tissue of obesogenic rats.

The morphometric analysis revealed a decrease in the area and perimeter of the hepatocytes and nuclei in the OD group, indicating a reduction in cell size. In contrast, a significant increase in the number of hepatocytes was observed in the OD group. This result suggests a possible picture of hepatocyte hyperplasia associated with liver regeneration due to diet. Hepatocyte hyperplasia may result from accelerated cell division in response to metabolic compensation due to the hepatic need, and this process is responsible for most of the mitoses observed [67,68]. Animal studies examining the effect of maternal obesogenic diets on the mother’s liver morphometry are still lacking in the literature. However, in a study by Altunkaynak and Ozbek [69], using non-pregnant rats fed a high-fat diet for three months, a decrease in the total number of hepatocytes was observed, along with a significant increase in the total number of binucleated hepatocytes in the group that received the high-fat diet.

In the present study, we verified the presence of binucleated hepatocytes in the OD group. This effect may be linked to an attempt at cellular regeneration, as the increase in binucleated hepatocytes is associated with the liver’s regenerative capacity in response to damage [70]. Furthermore, compensatory proliferation occurs in parallel with inflammatory processes that remove damaged hepatocytes and provide a repair process [71]. We observed an increase in pro-inflammatory cytokines in the liver of OD group, characterized by an increase in TNF-α and IL-1β. This increase may also be related to the proliferation of hepatocytes, as seen previously. TNF-α and IL-1β can promote DNA synthesis and hepatocyte proliferation, and their effects are mediated through the autocrine secretion of transforming growth factor alpha (TGF-α) from hepatocytes [72].

Pro-inflammatory cytokines, in turn, are known to increase ROS production and cell injury [73]. We observed that the rats in the OD group exhibited greater body adiposity and hepatic triglyceride accumulation, which contributed to the increase in these pro-inflammatory cytokines in the liver. In obesity, there is an increase in pro-inflammatory cytokines, such as TNF-α, which can lead to ROS accumulation, inducing the expression of pro-oxidant enzymes (e.g., NADPH oxidase) and reducing the expression of antioxidant enzymes (e.g., catalase) [74]. Furthermore, excess body adiposity can produce ROS, accompanied by elevated adipokine and TNF-α secretion, which promotes chronic inflammation [75]. While IL-1β stimulates hepatic fatty degeneration, it increases the precipitation of triglycerides and cholesterol in the liver parenchyma, leading to the formation of lipid droplets [76].

Oxidative stress is associated with the development of MASLD and plays a crucial role in the progression of hepatic steatosis [77]. When fatty acids are in excess or when there is difficulty in removing them, they can become a source of lipotoxic species that cause oxidative damage and liver injury [70]. Liver diseases are often associated with the accumulation of cellular injury due to the dysregulation of redox homeostasis, and this can occur when there is an increase in ROS production, such as superoxide (O_2_^−^) and hydrogen peroxide (H_2_O_2_), or a decrease in antioxidant enzyme activity, such as SOD, GPX, and catalase CAT [21]. This system’s disorder can lead to cellular oxidative stress, with a decrease in total thiols and an increase in carbonyl protein residues and derivatives of lipid peroxidation [78].

In the present study, we observed that rats fed with a maternal obesogenic diet presented oxidative stress in the liver, characterized by increased levels of MDA and carbonyls, decreased the activity of the SOD enzyme, in addition to a reduction in the cellular REDOX state (GSH/GSSG) and low levels of total thiols. Similar results were found by Rodríguez-González et al. [79], who demonstrated that rats fed a high-fat diet before mating and during pregnancy and lactation exhibited increased levels of carbonyls, with no change in MDA levels and SOD activity, but with increased GPx activity. Fisch et al. [24] also observed an increase in MDA levels and CAT enzyme activity, associated with a pro-inflammatory state due to increased levels of TNF-α in the liver in rodents fed a high-calorie diet during pregnancy and lactation. Candia et al. [80] reported in their work that pregnant mice fed an obesogenic diet for nine weeks before and during pregnancy exhibited a reduction in the antioxidant enzyme CAT and increased protein oxidation in the liver.

Our findings showed that the pro-oxidant state observed in rats fed an obesogenic maternal diet was not compensated by the reduction in endogenous antioxidant agents, suggesting a compromise in hepatic REDOX homeostasis. Furthermore, the maternal obesogenic diet administered for a short period (only during pregnancy and lactation) led to the accumulation of body and liver fat. This resulted in oxidative stress and an increase in pro-inflammatory markers, thereby predisposing to the development of liver diseases.

Therefore, this study in an animal model demonstrated that an obesogenic diet, when consumed in the short term (during pregnancy and lactation), causes significant metabolic alterations. This reinforces the need for clinical studies to evaluate how nutritional monitoring can prevent complications such as gestational diabetes, hypertension, and pre-eclampsia. These conditions, associated with the consumption of foods of low nutritional quality, affect maternal and fetal health and can lead to adverse perinatal outcomes and increase the risk of chronic diseases in the offspring. Understanding the effects of an obesogenic maternal diet is crucial for developing effective preventive strategies for maternal and child health.

Among the strengths of our study, we highlight the rapid induction of metabolic changes consistent with overweight and obesity in Wistar rats subjected to an obesogenic maternal diet. These findings validate the experimental model for investigating the effects of maternal nutrition on metabolic health. However, this study has limitations, as it lacks clinical data in humans and an assessment of the effects of the maternal diet on offspring. Therefore, future studies should include longitudinal investigations that integrate clinical and nutritional data in pregnant women. In this way, we can broaden our understanding of the long-term effects of an obesogenic diet on maternal health and the metabolic programming of offspring. Our research group is already conducting studies that evaluate the impact of intrauterine exposure to obesogenic diets on the metabolic health of offspring, contributing to our understanding of the role of maternal nutrition in fetal and postnatal development.

## 5. Conclusions

This study demonstrates that exposure to a diet high in fat and sugar, even during brief physiological periods such as pregnancy and lactation in rats, promotes increased body adiposity and predisposes to the development of maternal obesity. Additionally, the obesogenic diet led to hepatic metabolic disorders, resulting in an increase in pro-inflammatory cytokines and oxidative stress in the liver. These data are essential, since the rate of obesity has increased worldwide, including in women during pregnancy and postpartum, and this is associated with an inadequate diet during this period. The lipogenic effects of the maternal diet led to the accumulation of hepatic lipids and disrupted REDOX homeostasis, increasing the likelihood of developing diseases.

## Figures and Tables

**Table 1:** Composition of ingredients used in experimental diets.

Ingredient/Amount per 100 g of Diet	Vivarium Diet	Hyperlipidic Diet (g)	Sweetened Condensed Milk
Corn starch	-	11.8	-
Flour	-	12.0	-
Cornstarch biscuit	-	7.2	-
Soy flour	-	8.5	-
Lard	-	5.5	-
Margarine (65% lipids)	-	3.5	-
Milk cream (20% lipids)	-	3.0	-
Casein (>85%)	-	20.0	-
Sucrose	-	20.0	-
Soy oil	-	4.0	-
Cellulose	-	0.3	-
Mineral mix *	-	2.5	-
Vitamin mix **	-	0.7	-
DL-Methionine	-	0.3	-
Choline Bitartrate	-	0.25	-
BHT	-	0.0014	-
Sodium Chloride	-	0.36	0.026
Calcium	-	-	0.058
Total (g)	-	100	-
Kcal/100 g	3.44	4.52	304
% Total fat	10.9	31.99	17.7
% Proteins	28.3	20.18	9.8
% Carbohydrates	60.8	47.82	72.3

Source for type of diet: Vivarium diet; Hyperlipidic diet based on the study by Ferro Cavalcante et al. (2013) and Oliveira et al. (2025) [28,29], adapted from the Food Budget survey (POF) 2002/2003. Calculations based on nutritional composition information sent by the product supplier and Brazilian Table for Food Composition (TBCA). * Mineral Mix is composed by the reagents (in mg/kg of diet): CaHPO_4_, 17.20; KCl, 4000; NaCl, 4000; MgO, 420; MgSO_4_, 2000; Fe_2_O_2_, 120; FeSO_4_.7H_2_O, 200. ** The Vitamin Mix is composed by the reagents (in mg/kg of diet): Folic acid, 200; Nicotinic acid, 3000; Biotin, 20; BHT: Butylhydroxytoluene; Calcium Pantothenate, 1600; Pyridoxine.HCl, 700; Riboflavin, 600; Thiamine.HCl, 600; Vitamin A, 400,000 IU; Vitamin B12, 2500; Vitamin D3, 100,000IU; Vitamin E, 7500 IU; Vitamin K1, 7500Ul; sweetened condensed milk (Italac^→^), food composition was in accordance with the label.

**Table 2:** Primers used to PCR analysis.

Gene	Forward Primer (5′-3′)	Reverse Primer (5′-3′)
β2M	TGACCGTGATCTTTCTGGTG	ACTTGAATTTGGGGAGTTTTCTG
IL1β	GCCACCTTTTGACAGTGATG	CCCAGGTCAAAGGTTTGGA
TNFα	AGGCACTCCCCCAAAAGATG	TGAGGGTCTGGGCCATAGAA

β2M: beta 2-microglobulin, IL1β: Interleukin 1 Beta, TNFα: Tumor Necrosis Factor alpha.

**Table 3:** Weekly food consumption in kcal of rats during pregnancy.

		1° Week (kcal)	2° Week (kcal)	3° Week (kcal)	Total Period of Pregnancy (kcal)
	Ptna	154.4 ± 15.50	198.0 ± 15.65	198.2 ± 13.72	550.6 ± 43.39
	Lip	59.75 ± 5.99	76.63 ± 6.05	76.70 ± 5.31	213.1 ± 16.79
	CHO	331.7 ± 33.29	425.3 ± 33.61	407.1 ± 38.99	1164 ± 102.0
CD	Total	545.8 ± 54.78	699.9 ± 55.31	682.0 ± 56.57	1928 ± 161.7
	Ptna	96.87 ± 4.93 **	86.47 ± 4.51 ***	84.71 ± 3.44 ****	268.0 ± 11.71 ***
	Lip	159.0 ±8.18 ****	137.3 ± 6.08 ***	138.5 ± 5.53 ****	434.9 ± 18.36 ****
	CHO	392.3 ± 36.78	307.7 ± 23.06 *	285.4 ± 13.30 *	985.4 ± 69.83
OD	Total	648.2 ± 41.18	531.5 ± 31.11 *	508.6± 20.52 *	1688 ± 87.56

The difference between the OD group (*n* = 5) and the CD group (*n* = 5) was considered significant regarding the same week of food and macronutrient consumption (* *p* < 0.05; ** *p* < 0.01; *** *p* < 0.001; **** *p* < 0.0001). kcal = kilocalories; Ptna = proteins; Lip = lipids; CHO = carbohydrates.

**Table 4:** Weekly food consumption in kcal of rats during lactation.

		1° Week (kcal)	2° Week (kcal)	3° Week (kcal)	Total Period of Lactation (kcal)
	Ptna	247.6 ± 5.73	401.8 ± 7.41	375.4 ± 20.51	1025 ± 19.53
	Lip	95.82 ± 2.21	155.5 ±2.87	145.3 ± 7.93	396.6 ± 7.55
	CHO	531.8 ± 12.31	863.1 ± 15.93	806.4 ± 44.05	2201 ± 41.95
CD	Total	875.3 ± 20.26	1420 ± 26.23	1327 ± 72.49	3623 ± 69.03
	Ptna	84.02 ± 12.86 ****	153.9 ± 11.87 ****	154.1 ± 19.11 ****	392.0 ± 42.71 ****
	Lip	167.2 ± 21.54 *	252.2 ± 19.36 **	255.3 ± 30.22 **	674.7 ± 50.52 ***
	CHO	308.0 ± 37.82 ***	555.0 ± 40.90 ***	546.8 ± 39.15 **	1410 ± 108.4 ***
OD	Total	559.3 ± 52.29 ***	961.1 ± 72.05 ***	956.3 ± 84.91 *	2477 ± 194.0 ***

The difference between the OD group (*n* = 5) and the CD group (*n* = 5) was considered significant regarding the same week of food and macronutrient consumption (* *p* < 0.05; ** *p* < 0.01; *** *p* < 0.001; **** *p* < 0.0001). kcal = kilocalories; Ptna = proteins; Lip = lipids; CHO = carbohydrates.

**Table 5:** Analysis of the biochemical profile in the serum and liver tissue of rats fed with an obesogenic diet during pregnancy and lactation.

Serum Biochemical Analysis (mg/dL)	CD Group	OD Group	*p* Value
Glucose	106.3 ± 7.45	140.2 ± 10.25	*p* = 0.0200 *
Triglycerides	157.3 ± 19.67	283.3 ± 24.06	*p* = 0.0037 **
Total cholesterol	71.41 ± 7.26	92.40 ± 5.50	*p* = 0.0469 *
HDL cholesterol	0.0367 ± 0.0004	0.0342 ± 0.0006	*p* = 0.0080 **
**Biochemical analysis of liver tissue (mg/dL)**	**CD group**	**OD group**	***p* value**
Triglycerides	179.7 ± 8.96	237.6 ± 18.53	*p* = 0.0226 *
Total cholesterol	45.63 ± 3.57	61.41 ± 1.74	*p* = 0.0042 **

Analyzes were performed using Labtest^®^ colorimetric kits. *n:* 5–7 animals per group. * *p* < 0.05; ** *p* < 0.01. Groups: control diet group (CD); obesogenic diet group (OD).

## Data Availability

The data generated or analyzed during this study are provided in full in the published article. If requested by the journal, the gross values may be sent.
